# A hybrid IGRT workflow using SGRT and CBCT for prostate SBRT: Feasibility, efficiency, and safety

**DOI:** 10.1002/acm2.70339

**Published:** 2025-11-07

**Authors:** Juergen Meyer, Angelia Tran, Ting Martin Ma, Bing‐Hao Chiang, Tamara Egan, Jonathan J. Chen, Yinghua Tao, Ning Cao, Katherine H. Kim, Jay J. Liao, Sharareh Koufigar, Winston Vuong, Emily S. Weg

**Affiliations:** ^1^ Department of Radiation Oncology Fred Hutchinson Cancer Center University of Washington Seattle Washington USA; ^2^ Present address: Sutter Health Sansum Medical Group, 540 W Pueblo St Santa Barbara California USA; ^3^ Present address: Stich Radiation Center Weill Cornell Medicine New York USA

**Keywords:** image guided radiation therapy (IGRT) workflow optimization, patient monitoring, patient safety, prostate stereotactic body radiation therapy (SBRT), SGRT tolerances, surface guided radiotherapy (SGRT)

## Abstract

**Background and purpose:**

Safe delivery of prostate stereotactic body radiotherapy (SBRT) relies on precise target localization. Without access to real‐time intrafraction motion management, careful optimization of IGRT protocols is necessary to safeguard treatment accuracy and patient outcomes.

**Methods:**

An IGRT workflow is proposed that incorporates surface‐monitoring (SGRT) to complement cone‐beam CT (CBCT) imaging. The study evaluates 23 consecutive SBRT prostate patients who were treated on a prospective registry study. Each patient received pre‐ and mid‐treatment and a subset received post‐treatment CBCTs. The frequency and magnitude of SGRT triggered beam interruptions as well as treatment times were recorded.

**Results:**

The median number of CBCTs acquired per fraction was four and the median treatment time was 23 min (IQR 19–27). SGRT detected intra‐fraction surface‐based motion beyond a combined 4 mm vector isocenter tolerance in 62% of all fractions treated, with a maximum motion of 15 mm. On average < 2 beam interruptions were triggered by SGRT per treatment fraction. There was no statistically significant correlation between overall treatment time and SGRT‐triggered beam interruptions (*r* = 0.048, *p* = 0.645). There was a weak but statistically relevant correlation of overall treatment time with the maximum detected motion (*r* = 0.23, *p* = 0.026). SGRT detected five fractions where the patients had persistently moved outside the SGRT tolerance, and for three of these (60%), a CBCT verified that the target was out of tolerance.

**Conclusion:**

SGRT is a valuable tool that complements CBCT‐based IGRT. An SGRT motion vector tolerance of 4 mm provides a pragmatic compromise between detecting patient motion and treatment efficiency. Overall, persistent patient motion during treatment was infrequent in this cohort, however, SGRT was able to detect several cases where the internal target was outside of the tolerance highlighting that patient monitoring with SGRT can contribute to improved quality and safety for prostate SBRT.

## 1 INTRODUCTION

Prostate cancer remains one of the most prevalent malignancies affecting men worldwide. Stereotactic body radiation therapy (SBRT) has emerged as a promising treatment modality for localized prostate cancer with multiple prospective trials^1^, ^2^, ^3^ and large meta‐analyses^4^, ^5^, ^6^, ^7^, ^8^ demonstrating favorable tumor control outcomes with low rates of high‐grade toxicity. SBRT delivers highly focused, escalated doses of radiation over a shorter treatment course, typically five fractions or less.^9^ The success of SBRT in prostate cancer treatment depends not only on the prescribed dose and accurate targeting of the tumor, but also on the ability to account for the inherent *anatomical* and *physiological* variations, such as bladder and rectum filling^10^ that occur during treatment. Another important consideration is *patient motion* following initial image‐guided setup^11^, ^12^ which has been shown to increase with treatment duration in radiation therapy patients.^13^, ^14^, ^15^, ^16^

It has been demonstrated that larger intrafraction motion during SBRT is associated with increased toxicity and their occurrence among patients appears to be stochastic.^17^ Image‐guided radiation therapy (IGRT) has played a pivotal role in enhancing the precision of prostate SBRT^18^, ^19^, ^20^, ^21^ by providing imaging and correction capabilities, allowing for the precise alignment of the treatment beam with the tumor target. A recent survey by the NRG Oncology Medical Physics Subcommittee^22^ showed that 59% of respondents used in‐room cone beam computed tomography (CBCT), 23% used 2D kV or port imaging and 3% radio marker for the main position correction/verification for prostate SBRT, with volumetric arc therapy (VMAT) being the dominant delivery method at 68%. Despite the availability of real‐time imaging on MRI‐linacs^23^, ^24^ and surrogate tracking of implanted markers using intrafraction kV imaging^25^ or electromagnetic beacons^26^, ^27^ many clinics do not have real‐time capabilities to monitor intrafraction target motion. Instead, mid‐treatment CBCT may be acquired to obtain a single snapshot of the internal anatomy at the halfway point, so that corrective action can be taken if any movement of the target or relevant organs‐at‐risk (OARs) is detected.

Recently, surface‐guided radiation therapy (SGRT) has emerged as an additional technology^28^, ^29^, ^30^, ^31^ to improve the quality and safety of radiotherapy treatments.^32^, ^33^ SGRT utilizes non‐invasive optical imaging and tracking to continuously monitor the patient's external surface during treatment, providing valuable information about patient positioning and motion in real‐time. Unlike traditional internal IGRT methods, SGRT offers the advantage of no additional radiation exposure to the patient while providing continuous monitoring capabilities of the patient's surface. The time dependence of patient motion is well documented,^14^ however, it is typically small compared to conventional treatment margins if treatment is completed within a 15‐min window. For prostate SBRT, treatment margins are smaller, and treatment durations are typically longer when conventional dose rates are used. Although adding a mid‐treatment CBCT provides a valuable checkpoint to verify target and OAR positions, the acquisition and image review also increases the overall treatment duration.

The amount and frequency of patient motion and how much it contributes to prostate motion is not well understood. The intra‐fractional prostate motion reported in the literature typically does not separate actual prostate motion from patient motion.^34^ Although SGRT cannot detect internal variations,^11^ any posture or positional changes can trigger a beam hold. The integration of SGRT with IGRT in prostate SBRT treatments has the potential to enhance targeting accuracy and precision. A combined approach leverages the benefits of both technologies, with x‐ray based IGRT ensuring accurate alignment of the internal target, while SGRT provides immediate feedback on external patient motion and can be sensitive to large changes in bladder filling as this can change the shape and position of the abdominal and pelvic surface. Consequently, although less effective than internal motion tracking when used alone, an integrated approach may enhance patient safety compared to having no continuous monitoring.

There are no consensus recommendations on what SGRT motion tolerance is appropriate for prostate SBRT treatments for the different SGRT systems since they use different parameters and surface registration algorithms. We explored the evolving role of SGRT as a complementary tool to IGRT in prostate SBRT and investigated how to best implement it alongside volumetric imaging in the clinical workflow. Furthermore, we evaluated the potential advantages and challenges associated with this combined approach, with a focus on its impact on treatment efficiency and patient safety.

## 2 MATERIALS AND METHODS

An institutional prospective prostate SBRT registry study (IRB STUDY ID: STUDY00014709, MOD ID: MOD00018067) was opened in 2022. Here, we report on the first 23 consecutively treated patients diagnosed with localized prostate cancer treated with SBRT. Patients were excluded if they required pelvic nodal irradiation or had prior transurethral resection of the prostate (TURP). Patients were selected based on the following eligibility criteria. Localized intermediate risk prostate cancer with a prostate measuring below 60 cc, an International Prostate Symptom Score (IPSS) below 21, and no posterior extracapsular extension that would preclude placement of a rectal spacer. Patients had either SpaceOAR or the radiopaque SpaceOAR Vue hydrogel (Boston Scientific, Marlborough, MA) injected between the prostate and rectum prior to their simulation appointment. Prior to treatment, all patients had three cylindrical gold fiducials (0.8 mm × 5 mm), implanted for better localization of the prostate during treatment. Patients were instructed to void their bladder and drink 16 oz (473 mL) of water 45 min before each treatment. A daily pre‐treatment bladder ultrasound was conducted to confirm bladder filling. Target bladder volumes were typically 150–250 cc. For rectal emptying, patients did an enema 2–3 h before each fraction. Computed tomography (CT) scans were obtained with 1.25 mm slice thickness, and a non‐contrast magnetic resonance imaging (MRI) scan was obtained on the day of simulation and fused with CT to facilitate accurate target delineation. Patients were immobilized in custom vacuum bags (Klarity, Heath, OH) and received three tattoos for setup.

The clinical target volume (CTV) encompassed the prostate gland and the proximal 1 cm of the seminal vesicles (SV), and the planning target volume (PTV) was generated with a 5 mm radial and 3 mm posterior expansion from the CTV to account for setup uncertainties. Some patients had a separate CTV contoured for the SV, with a 5 mm radial expansion to a lower dose PTV. OARs were initially contoured with Limbus AI (Limbus AI Inc., Canada) auto‐contouring^35^ and finalized and approved by the attending physician. A uniform 3 mm expansion around the fiducials was added to aid with image guidance during review of the CBCTs. Dose constraints were defined based on institutional guidelines and recommendations of the radiation therapy oncology group (RTOG) 0938 protocol, AAPM Task Group 101,^36^ and a prospective single‐institution dose escalation study of prostate SBRT.^4^ 20 patients received 40 Gy to the PTV delivered in five fractions and either 30 or 40 Gy to the SV. Three patients received 35 Gy in five fractions. All patients were planned with the Monaco treatment planning system (Elekta, Crawley, UK) for Elekta linacs with Agility multileaf collimators operated at standard dose rate. Plans used two volumetric arc therapy (VMAT) beams with 6 or 10 MV photons and calculated with Monte Carlo. Tattoos were used for initial setup to room lasers.

**FIGURE 1:**
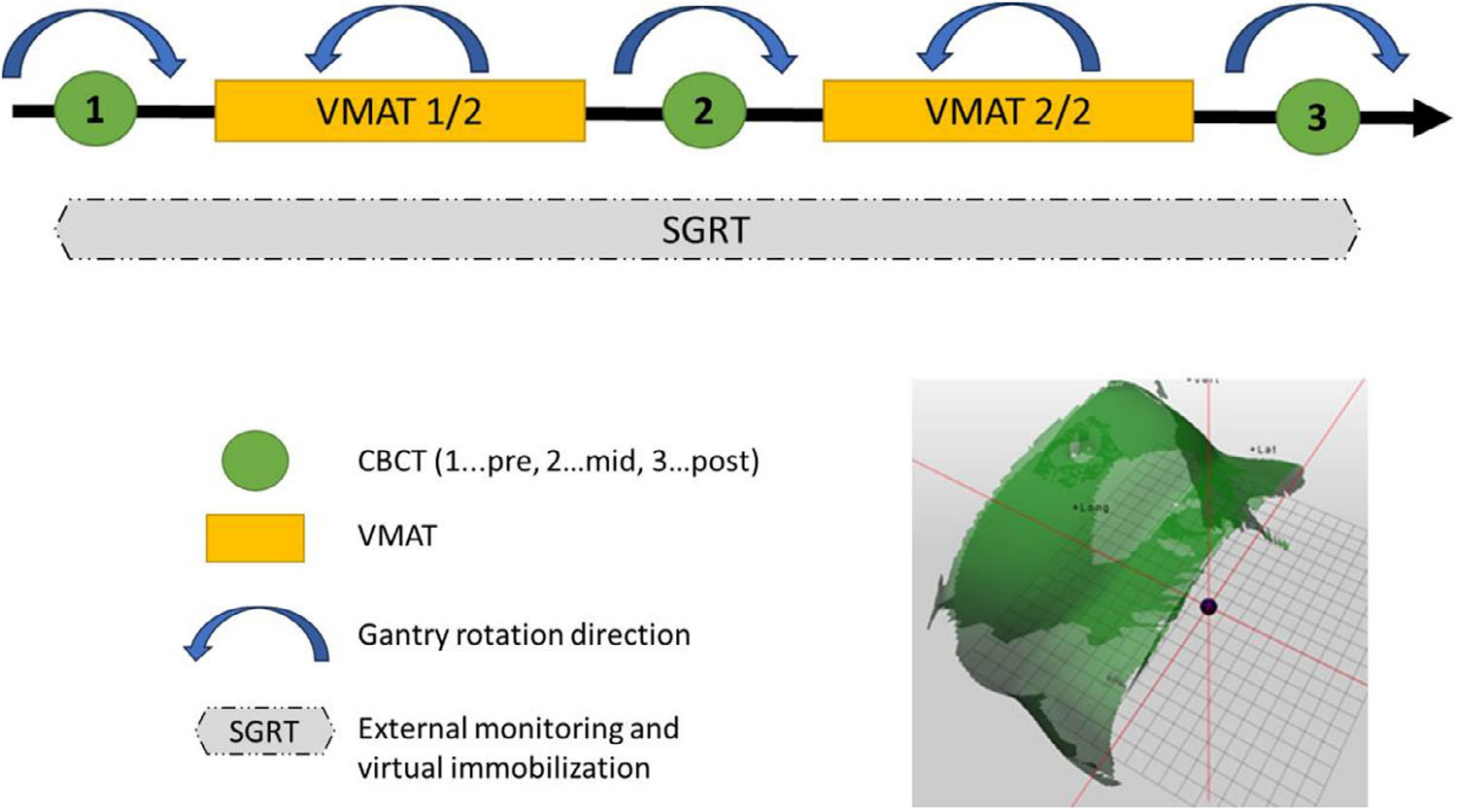
Schema for prostate SBRT IGRT and delivery. A representative SGRT reference surface is shown at the bottom right.

The IGRT workflow is shown in Figure 1. Patients first received a CBCT (XVI version 5.0.7.1) in a clockwise direction. Shifts were obtained based on an initial automatic grey value match of a preselected region of interest, which was then manually adjusted to match the gold fiducials within the IGRT margin and additional personalized IGRT instructions by the attending physician. Only translational errors were applied and a new SGRT reference surface was captured for the current fraction with C‐RAD (C‐RAD, Uppsala, Sweden) as highlighted in Figure 1. If the shifts based on the CBCT were > 3 mm in any direction, an additional verification CBCT was acquired after the shifts had been applied and prior to capturing a new SGRT reference. The reference surface covered the pelvic region as illustrated in Figure 1. The C‐RAD system was operated in *cMotion* monitoring mode and connected to the Elekta Response^TM^ gating interface so that a beam interruption was automatically triggered when the patient position was outside of an SGRT tolerance of 4 mm. The response time to turn the beam off has been reported to be < 1s.^37^ Our empirically derived 4 mm tolerance represents the combined 3D vector of the three translational errors as calculated by C‐RAD. In a comparable study which also utilized C‐RAD, a slightly larger tolerance of 5 mm^38^ was used. It is of note that a value of 4 mm appears larger than what has been reported in other studies, e.g. Macedo‐Jiménez et al^11^ reported a tolerance of ± 3 mm in each translational direction. A more detailed analysis reveals a 4 mm tolerance for C‐RAD is in fact tighter, as a ± 3 mm tolerance in each direction results in a 3D vector of 5.2 mm, which makes it comparable to the 5 mm tolerance used by Mannerberg et al.^38^

If at any point during treatment a patient persistently stayed outside the SRGT tolerance, an additional CBCT was acquired. As a point of reference, ‘persistent’ was defined as > 30s, but some flexibility was allowed based on the treating therapists’ discretion. The first VMAT arc was delivered in counterclockwise direction to minimize unnecessary gantry rotations. Next, a mid‐treatment CBCT was acquired to ensure the prostate had not moved out of tolerance. Per IGRT protocol, any shifts were applied and a new SGRT reference surface was captured for patient monitoring. Corrections larger than 3 mm were verified with an additional CBCT. After delivery of the second VMAT arc, a post‐treatment CBCT was obtained when it was feasible to assess for prostate movement during the second half of the treatment.

Parameters evaluated were the total treatment time, the number of SGRT triggered beam interruptions and maximum shifts detected as well at the number of CBCTs and shifts associated with the different timepoints.

## 3 RESULTS

**FIGURE 2:**
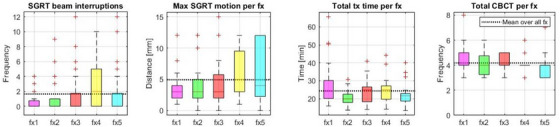
Per fraction analysis of all patients (from left to right): boxplots of a) SGRT‐triggered beam interruptions, b) maximum motion detected by SGRT, c) total treatment time, and d) the total number of CBCTs.

The mean age of patients included was 68.4 ± 8.0 years, the median PSA level was 6.6 ng/mL (IQR:4.7–7.5) and Gleason Score 7. In Figure 2, a per fraction analysis is shown averaged over all patients. On average SGRT stopped the beam two times per treatment fraction. The average maximum SGRT detected surface motion was 5 mm and a maximum of 15 mm with a trend towards larger maximum surface motion as the fraction number increased. The average total treatment time was 23 min (IQR 19–27) and the average number of CBCTs was four.

**FIGURE 3:**
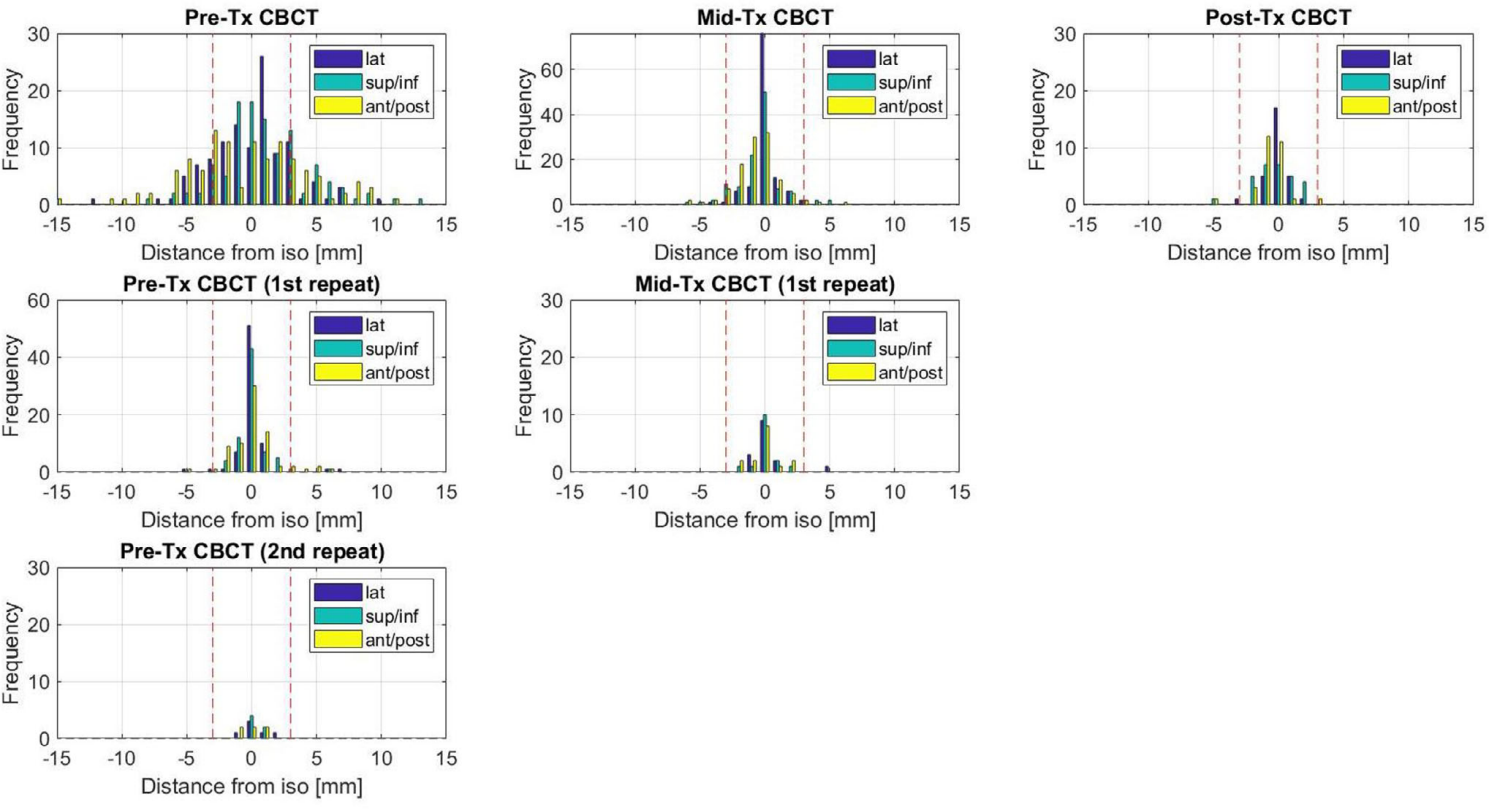
Shift distribution after CBCTs of all patients at the different time points during treatment.

The collective shift distributions based on CBCTs are shown in Figure 3. 64% of the pre‐treatment CBCT had shifts > 3 mm in at least one direction triggering a verification CBCT after the shifts were applied. The dominant shifts were in superior/inferior (sup/inf) and anterior/posterior (ant/post) direction. The lateral (lat) shifts were generally of smaller magnitude. In 8% of the verification CBCTs a shift > 3 mm remained requiring a second verification scan after the shifts had been applied. After the 2nd verification scan, all shifts were < 3 mm. For the mid‐treatment CBCT, 13% of patients had shifts > 3 mm with the majority of those being in longitudinal and anterior/ posterior direction. This agrees with what has been reported in the literature.^36^ The mid‐treatment verification scan only showed one case outside of the 3 mm tolerance. For the post treatment CBCT, only one patient had shifts > 3 mm with two directions being off by 5 and 3 mm. The patient set up initially with < 2 mm shifts after the pre‐treatment CBCT but had a 5 mm shift during the mid‐treatment CBCT in the opposite direction to the post treatment CBCT, indicating the prostate had moved laterally and then back to its initial position. It should be noted that only 25% (29/115) of the post‐treatment CBCTs could be analyzed due to a) data loss after a software upgrade, b) the inability to perform a registration retrospectively in the oncology information system (OIS) if it had not been done at the treatment console and c) the fact that a post‐treatment CBCT was not acquired for every patient if it impacted patient waiting times after machine repairs and other unforeseen delays.

**FIGURE 4:**
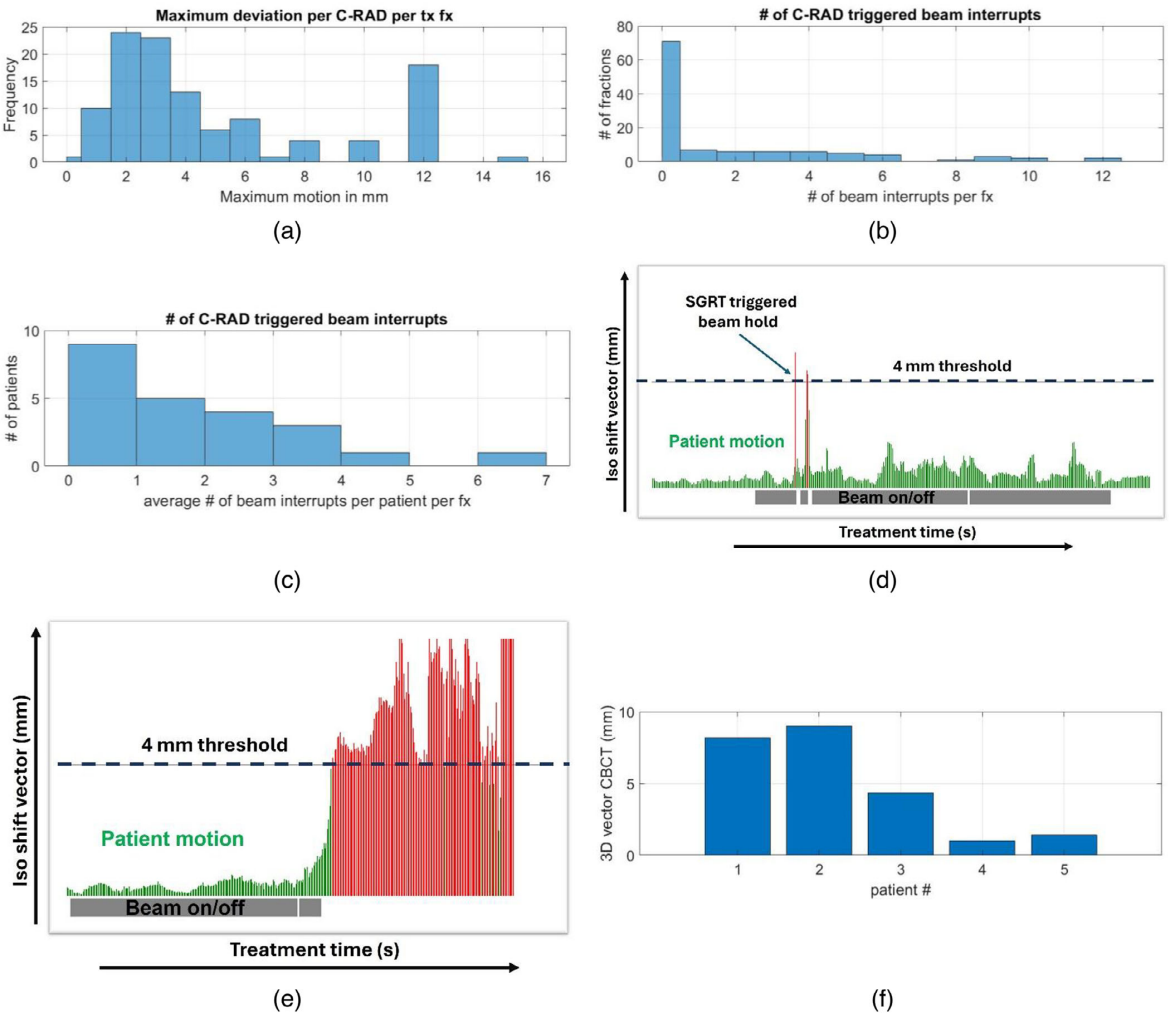
SGRT interventions. a) Distribution of maximum patient motion detected per treatment fraction, b) distribution of the number of beam interrupts triggered by SGRT over all fractions, c) average number of beam interrupts per patient per treatment fraction, d) illustration of two short beam interrupts triggered by SGRT‐ red represents patient motion beyond tolerance, grey bars denoting beam on, e) long beam interruption indication patient remained out of tolerance, and f) 3D vector of translational errors after SGRT‐triggered CBCT, as quantified on CBCT.

The impact of SGRT‐related interventions is shown in Figure 4. The maximum shift suggested by SGRT per fraction for all treatments is shown in Figure 4a, with a maximum value of 15 mm. A shift > 4 mm was set to trigger a beam interruption per imaging protocol. This value represents the 3D vector of the three translational correction parameters. An SGRT‐triggered beam interruption happened in 62% (71/114) fractions. In Figure 4b the distribution of the number of beam interruptions per fraction is shown, and Figure 4c shows the number of beam interruptions per patient per fraction showing that 91% (21/23) of patients had on average four or fewer beam interruptions triggered by SGRT. Most beam interruptions were for a short duration, in the order of a few seconds, and treatment was continued automatically after the patient's position returned within tolerance, as illustrated in Figure 4d. However, during five fractions SGRT indicated that the patient had moved and stayed outside the 4 mm tolerance persistently (illustrated in Figure 4e), triggering an additional CBCT to verify the internal anatomy and positioning of the target volume. Two of those five fractions were for the same patient. The 3D vectors of errors based on CBCT are shown in Figure 4f. In two of those scenarios the resulting 3D vectors were ≤1 mm and treatment could continue without further intervention as the changes in the surface information did not translate to the internal anatomy.

**FIGURE 5:**
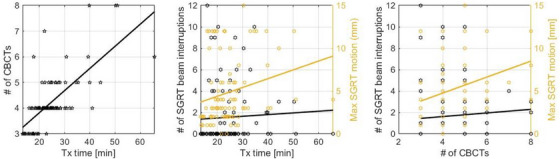
Correlations between SGRT, number of CBCTs and treatment time. a) Treatment time versus the number of CBCTs, b) treatment time versus SGRT‐triggered beam interruptions (primary axis) and maximum detected motion detected by SGRT (secondary axis), and c) correlation between the number of CBCTs and SGRT‐triggered beam interruptions (primary axis) and maximum detected motion detected by SGRT (secondary axis). Trendlines represent linear fits to the data.

The correlation between treatment time, SGRT, and the number of CBCTs is shown in Figure 5. A linear fit to the data in the scatter plot is shown to visualize trends. The Pearson's correlation coefficient was calculated to measure correlation between the various relationships. A statistically significant positive correlation was found between the treatment time and the number of CBCTs taken, *r*(92) = 0.70, *p* < 0.001, with 0.05 significance level. No statistically relevant correlation was found between the treatment time and SGRT‐triggered beam interruptions, *r*(92) = 0.05, *p* = 0.645 and the number of CBCTs and SGRT‐triggered beam interruptions, *r*(114) = 0.06, *p* = 0.518. A weak but statistically relevant correlation was found between the treatment time and the maximum motion detected by SGRT, *r*(92) = 0.23, *p* = 0.026 and between the number of CBCTs and SGRT‐triggered beam interruptions and the maximum motion detected by SGRT, *r*(114) = 0.25, *p* = 0.007.

## 4 DISCUSSION

The National Comprehensive Cancer Network has endorsed SBRT as a standard‐of‐care treatment option for localized prostate cancer of all risk categories.^39^ Compared to conventionally fractionated radiation therapy, where each treatment fraction contributes only around 2% of the total dose, geometrical miss due to intra‐fraction motion in SBRT has a much larger impact given far fewer fractions. With specialized treatment machines, such as an MRI‐linac,^40^ daily adaptation,^41^ and real‐time tracking^42^, ^43^, ^44^, ^45^ of anatomical or positional changes become feasible. However, in the absence of real‐time imaging capabilities and fast delivery, robust image guidance strategies are required to ensure safe delivery. A recent practice survey^22^ showed that the majority of clinics use CBCT guidance when delivering prostate SBRT. It is not recommended to solely use SGRT for prostate treatments due to the poor correlation between external and internal anatomy.^11^, ^46^

In this work, SGRT is employed as a complementary system in addition to image‐guidance with CBCT as it has the ability to promptly detect external positional changes with submillimeter accuracy^47^ thus adding an additional layer of safety.^32^ The overall aim of this work was to assess the utility of SGRT for prostate SBRT treatments in terms of detecting patient motion. The secondary aim was to evaluate the efficacy of our empirically derived SGRT tolerance in an integrated IGRT workflow to explore and optimize the synergy between x‐ray‐based internal CBCT imaging and optical external imaging. We were interested in evaluating and tailoring our SGRT approach specifically for our prostate SBRT workflow with conventional dose rate delivery to optimize both treatment efficiency and patient safety.

From a delivery time point of view, the utilization of flattening filter free (FFF) beams is preferred due to the faster dose rates achievable, resulting in reduced delivery times on the order of 50%.^48^ Unfortunately, not much information is available in terms of utilization rates of FFF beams in general and for prostate SBRT in particular. However, a national survey in Japan conducted in 2019 found that, overall, 28% had adopted FFF beams, but uptake was strongly vendor dependent and below 15% for one of the two main vendors.^49^ Also, some modern ring‐gantry based systems do not offer high dose rate beams and treatment times of up to 60 min have been reported.^11^ Although it is expected that the uptake of FFF beams has increased, given that the typical lifetime of a linac is 10–15 years it is likely that many clinics still treat with conventional dose rates and do not have continuous intrafraction imaging capabilities and could benefit from SGRT. For context, although MRI‐guided linacs offer intrafraction imaging and at least one vendor includes FFF beam capability, their maximum dose rates, around 6 Gy/min^50^ and ∼4 Gy/min^51^ remain comparable to those of conventional systems.

For the 23 patients included here the average number of beam interruptions per treatment fraction over all patients and all five fractions was 1.6 (Figure 2) and average maximum motion was 4.9 mm. Most patients had less than 2 beam interruptions per treatment fraction with several outliers that had up to 12. Given that the SGRT reference is recaptured after each CBCT a new motion reference was captured at least twice during each fraction. Thus, strictly speaking the numbers do not reflect the overall patient motion per fraction but the motion between CBCTs. That means that with two arcs with corresponding CBCTs as a minimum per treatment, on average there was < 1 beam interruption per arc. The increase in the median and 25th percentiles for fraction 4 appears to be random and not systematic in nature. The median treatment time per fraction was 23 min (IQR 19–27) with an average of 24 min and 20s, which was reasonably consistent across all fractions and so was the total number of CBCTs with an average of 4.2 CBCTs per fraction.

The scatter plots in Figure 5 show a clear correlation between treatment time and the number of CBCTs. No statistically significant correlation was found between treatment time and SGRT‐triggered beam interruptions. However, since the surface tracking is reset after each CBCT, the actual tracking time is shorter compared to the overall treatment time and on average falls below the 15 min window beyond which prostate motion has been reported to increase.^14^ By recapturing a new tracking reference after CBCT, it is ensured that before the beam gets turned on tracking starts at zero rather than the edge of the range thereby avoiding unnecessary beam interruptions if motion is less than 4 mm. The maximum detected motion on the other hand is more strongly correlated with time indicating that on average for every 10 min extra time the maximum motion increases by approximately 1 mm. This is a relatively weak correlation although statistically significant.

We empirically chose a 4 mm SGRT 3D vector threshold in accordance with our IGRT strategy. It was found that in approximately two thirds (64%) of all fractions > 3 mm correction was needed after the initial pre‐treatment CBCT. The value of the 1st verification CBCT after the shifts have been applied rapidly diminishes, which is evidenced by the fact that 8% had remaining shifts with a magnitude of > 3 mm in at least one direction. Similarly, for the mid‐treatment CBCT, 13% had shifts with a magnitude of > 3 mm in at least one direction. It should be noted that all shifts were applied, even if < 3 mm, but only the ones > 3 mm triggered a verification CBCT. The diminished value of the re‐CBCT for the mid‐treatment scan is shown in Figure 3, where only one case had persistent shift parameters > 3 mm in one direction. This similarly applies to the post treatment CBCT. From an efficiency point of view this suggests that one verification CBCT after the pre‐treatment CBCT is useful but a verification scan after the first mid‐treatment CBCT might not be necessary. The same applies for the post‐treatment scans which were acquired as part of this study but were not intended to be used routinely. A pragmatic compromise would be to relax the re‐CBCT imaging tolerance from 3 to 5 mm after the first pre‐treatment CBCT and for the mid‐treatment CBCT thereby ensuring that if substantial changes are detected a verification scan is still carried out since there is a chance that intrafraction motion is still in process. This would not only reduce the imaging dose to the patient but also speed up the treatment without reduction in treatment accuracy and patient safety. Gorovets et al.^52^ showed that the probability of prostate motion increased with time, providing also additional motivation to use e.g. flattening filter free beams^53^ to minimize the overall treatment time.

The results in Figure 4 indicate that a 4 mm threshold for the C‐RAD SGRT system is a reasonable compromise between sensitivity and specificity. It is interesting to note that the maximum positional errors detected by SGRT amount to as much as 15 mm and that deviations > 4 mm were detected in 62% of all fractions. This information has previously not been available and strongly supports the use of SGRT for SBRT treatments even though the ‘excursions’ were mostly of short nature and the persistent errors translated in only 60% of the cases to the target volume, as shown in Figure 4f. However, Figure 4 highlights the importance of positional monitoring during SBRT as instances do occur where patients do not just temporarily move beyond treatment margins leading to geometrical miss. Despite the considerable number of SGRT‐triggered beam interruptions it was found that they are random in nature and typically short in duration and do not meaningfully impact the overall treatment time supporting the use of SGRT for prostate SBRT.

This study exhibits some limitations, which warrant consideration. One constraint is the sample size, consisting of 23 patients. We acknowledge that this subset may not accurately reflect a larger population. Not all post‐treatment CBCT's were accessible for analysis. Consequently, the findings presented in Figure 3, depicting the post‐treatment shift distribution, may not be fully representative of the entire patient cohort included in this study. In the context of patient monitoring, we observed some variability in the size of the SGRT reference surface in the superior/inferior direction and its positioning relative to the treatment isocenter.^47^ Although these variations may have had some influence on the tracking results, the overall impact is considered to be minimal. Data extraction from C‐RAD was limited to the magnitude of the detected patient motion, although internally all six degrees of freedom are monitored. This prevented us from doing a more detailed analysis comparing shift parameters between CBCT and SGRT. Although this would have been interesting to evaluate, it is already well known that external motion does not correlate well with internal anatomical changes during prostate treatments.^11^, ^46^ Future work in this registry study should encompass an analysis of patient outcome data, seeking correlations with the motion data. This will enhance our understanding of the broader implications and significance of the observed trends, providing a more nuanced and holistic perspective on outcomes.

## 5 CONCLUSIONS

An optimized IGRT protocol incorporating SGRT for additional real‐time monitoring has been proposed and implemented. The proposed SGRT threshold of 4 mm provided a practical balance between sensitivity to detect surface motion and treatment efficiency. SGRT detected positional changes above our SGRT threshold in 62% of the fractions with a maximum error of 15 mm and found several cases where the patients had moved persistently out of position, which would have led to a geometrical miss if it they not been detected by SGRT. This work aids in the optimization of IGRT imaging protocols for linac based prostate SBRT and demonstrates the feasibility of effectively incorporating SGRT to improve patient safety.

## AUTHOR CONTRIBUTIONS

The treating physicians Emily S Weg, Ting Martin Ma, Jonathan J Chen, Katherine H. Kim, Jay J Liao, and Winston Vuong were responsible for patient enrollment, treatment planning oversight, clinical decision‐making, and supervision of SBRT delivery. They ensured adherence to clinical standards throughout the study and provided critical review and interpretation of clinical data. Juergen Meyer, Angelia Landers, and Emily S Weg conceptualized the combined imaging protocol, conducted the quantitative analysis of motion data, contributed to statistical evaluation, and led the manuscript preparation and technical review. Bing‐Hao Chiang, Yinghua Tao, Ning Cao, and Sharareh Koufigar contributed to the oversight and validation of data collection, assisted in the analysis and interpretation of motion and imaging data. They also provided expert review of the methodology and results and contributed to the critical revision of the manuscript. Tamara Egan was responsible for coordinating treatment delivery and ensuring adherence to the image‐guided workflow. They provided practical insights into treatment efficiency and patient setup, supported data collection related to beam interruptions and motion events, and contributed to the review of the manuscript with a focus on workflow feasibility and clinical applicability.

## CONFLICT OF INTEREST STATEMENT

The authors declare no conflicts of interest.
